# Can chimerism explain breast/ovarian cancers in *BRCA* non-carriers from *BRCA*-positive families?

**DOI:** 10.1371/journal.pone.0195497

**Published:** 2018-04-16

**Authors:** Rachel Mitchell, Lela Buckingham, Melody Cobleigh, Jacob Rotmensch, Kelly Burgess, Lydia Usha

**Affiliations:** 1 Department of Internal Medicine, Division of Hematology, Oncology and Cell Therapy, Rush University Medical Center, Chicago, Illinois, United States of America; 2 Department of Pathology, Division of Medical Laboratory Science, Rush University Medical Center, Chicago, Illinois, United States of America; 3 Department of Obstetrics and Gynecology, Division of Gynecologic, Rush University Medical Center, Chicago, Illinois, United States of America; CNR, ITALY

## Abstract

Hereditary breast and ovarian cancer syndrome (HBOC) is most frequently caused by mutations in *BRCA1* or *BRCA2* (in short, *BRCA*) genes. The incidence of hereditary breast and ovarian cancer in relatives of *BRCA* mutation carriers who test negative for the familial mutation (non-carriers) may be increased. However, the data is controversial, and at this time, these individuals are recommended the same cancer surveillance as general population. One possible explanation for *BRCA* phenocopies (close relatives of *BRCA* carriers who have developed cancer consistent with HBOC but tested negative for a familial mutation) is natural chimerism where lack of detectable mutation in blood may not rule out the presence of the mutation in the other tissues. To test this hypothesis, archival tumor tissue from eleven *BRCA* phenocopies was investigated. DNA from the tumor tissue was analyzed using sequence-specific PCR, capillary electrophoresis, and pyrosequencing. The familial mutations were originally detected in the patients’ first-degree relatives by commercial testing. The same testing detected no mutations in the blood of the patients under study. The test methods targeted only the known familial mutation in the tumor tissue. Tumor diagnoses included breast, ovarian, endometrial and primary peritoneal carcinoma. None of the familial mutations were found in the tumor samples tested. These results do not support, but do not completely exclude, the possibility of chimerism in these patients. Further studies with comprehensive sequence analysis in a larger patient group are warranted as a chimeric state would further refine the predictive value of genetic testing to include *BRCA* phenocopies.

## Introduction

Germline mutations in *BRCA1* or *BRCA2* genes cause over 90% of hereditary breast and ovarian cancer (HBOC) syndrome [1–3]. *BRCA1* and *BRCA2* proteins play a critical role in repair of double-stranded DNA breaks and the maintenance of the genome integrity [3, 4]. Gene mutations result primarily in female cancers and an estrogen effect on tissue susceptibility has been postulated [5–7]. Truncating germline *BRCA1* and *BRCA2* (g*BRCA*) mutations are called deleterious or pathogenic and confer an extremely high risk of cancer. The lifetime risk of breast cancer in female g*BRCA* mutation carriers is up to 85%; the lifetime risk of ovarian cancer is up to 50% [8–16]. Some data suggest that the risk of endometrial cancer is also increased in g*BRCA* mutation carriers [17], especially, the risk of uterine papillary-serous carcinoma. If a woman previously diagnosed with breast cancer has a g*BRCA* mutation, she has up to a 65% risk of another breast cancer and a 16% lifetime risk of ovarian cancer [18, 19]. Female g*BRCA* mutation carriers typically present with breast cancer under age 50, but may also present with ovarian, fallopian tube, primary peritoneal, and pancreatic cancer occurring at any age. Because the risk of cancer is so high, it is recommended that these women undergo prophylactic surgery (removal of the breasts, fallopian tubes, and ovaries). If a woman declines surgery, it is recommended to undergo increased surveillance for breast and ovarian cancers which is, however, not as effective as prophylactic surgery at reducing cancer risk and may be not effective at all for ovarian cancer [20–25].

HBOC is inherited in an autosomal dominant pattern, meaning that affected individuals are heterozygous for g*BRCA* mutations. Patients suspected of carrying g*BRCA* mutations based on personal and family histories are recommended to undergo a test involving isolation of DNA from their white blood cells (or saliva) and sequencing of their *BRCA1* and *BRCA2* genes with deletion/duplication analyses.

If a relative of a g*BRCA* carrier has a blood test that is negative for the known familial g*BRCA* mutation, that individual is deemed to have a normal (wild-type) germline *BRCA1* and *BRCA2* genes and is often referred to as “*BRCA* non-carrier” [26]. Generally, there is no recommendation to undergo prophylactic surgery or increased surveillance for cancer. However, some *BRCA* non-carriers do develop breast or ovarian cancers. These individuals are referred to as “*BRCA* phenocopies,” meaning that they have the same phenotype (affected by an HBOC-associated cancer) as their relative, but do not have the same genotype (the known g*BRCA* mutation as shown by blood testing). Studies report conflicting results on the relative risk ratio (RR) of breast and ovarian cancers in *BRCA* non-carriers with the breast cancer RR up to 5.1 [27, 28]. Some authors argue that their cancer risk is the same as in the general population [29, 30], some conclude that their risk is the same as in high risk families without identified g*BRCA* mutations [26], but overall most authors agree that their risk is increased [31, 32]. The studies are difficult to compare because they use different methods and are applied to different populations [33]. Currently, the only explanations offered to *BRCA* phenocopies on the cause of their cancers are: 1) their cancers are sporadic; 2) they may have germline mutations in other genes that cause HBOC which have not yet been identified; 3) there are familial environmental factors that lead to their cancer. All of these explanations assume that cancers in *BRCA* non-carriers are not related to the familial g*BRCA* mutation. The risk of *BRCA* non-carriers developing an HBOC is clinically important because it determines their cancer surveillance and prevention recommendations [34].

**Fig 1:**
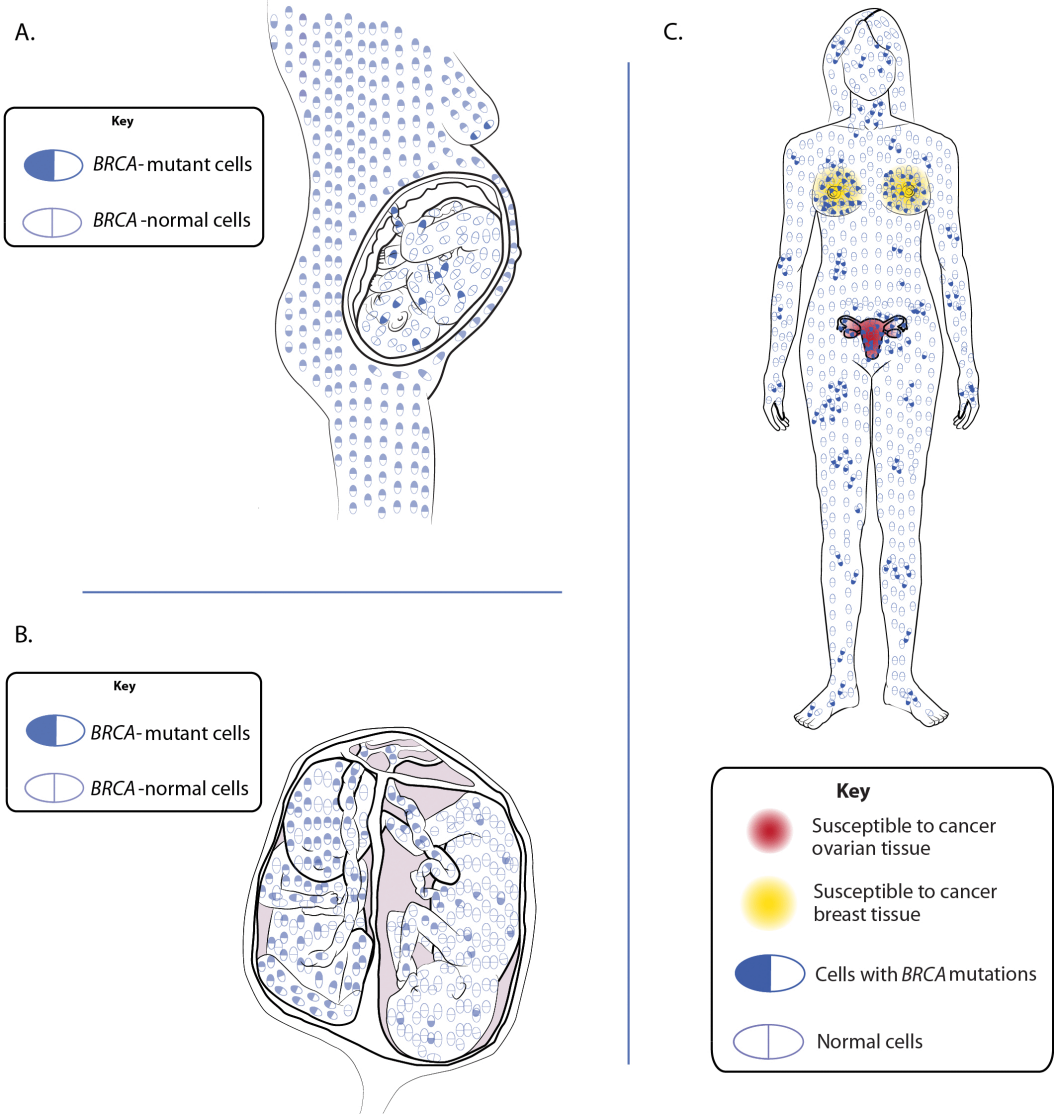
*BRCA* phenocopy hypothesis. A. Maternal-fetal microchimerism. B. Tetragametic chimerism. C. A woman with *BRCA*-mutant chimeric cells.

An alternate explanation for *BRCA* phenocopies that we further explored was natural chimerism. We hypothesized that in at least some *BRCA* phenocopies, breast and ovarian cancer are still caused by familial *BRCA* mutations, but the mutant genes are transmitted through an alternative non-mendelian inheritance, via chimeric cells harboring the mutation rather than through the germline [35, 36]. We further hypothesized that it is these *BRCA-*mutant cells that give rise to breast and ovarian cancers in chimeric individuals as these cells are known to be susceptible to malignant transformation. See Fig 1A. Therefore, the progeny of these cells (the majority of cells in the tumor) would be *BRCA*-mutant as well. This hypothesis gave us a potential explanation for the existence of *BRCA* phenocopies but also, the method to test the hypothesis by analyzing the patient’s tumor tissue for the known familial mutation. Finding the familial mutation in the patient’s tumor would have no other explanation except for chimerism. Even though somatic *BRCA* mutations are common in breast and especially, in ovarian cancer, it would be highly improbable to find the same somatic mutation in the patient’s tumor as the germline mutation present in her first-degree relative.

Chimeric or mosaic individuals harbor two different cell populations with different genetic compositions, arising from two different zygotes. For our purposes, “chimeric” will be preferred to “mosaic” as “mosaic” usually refers to cells arising from the same zygote with an acquired mutation in a daughter population.

Chimerism in animals was first reported in 1917 [37]. Human chimerism has been observed in naturally-occurring instances as well as iatrogenic cases (such as transplant recipients and rarely, following blood transfusions) [38]. Here, we focus on naturally-occurring chimerism which is a result of pregnancy.

Several types of naturally-occurring chimerism have been described in humans including tetragametic chimerism (TGC), fetal-maternal microchimerism (FMMc) and maternal-fetal microchimerism (MFMc) [39] (Fig 1). TGC has been observed in twin pregnancies. Occasionally, a twin may die early in pregnancy (“vanishing twin") resulting in the singleton birth; however, his/her cells may be admixed to the body of the surviving twin [40]. The rate of twinning and chimerism was reported to increase with the wide use of assisted reproduction techniques [41–46]. In contrast to TGC, FMMc and MFMc are thought to be quite common if not ubiquitous. These events could lead to a chimeric individual with tissues of different genotype. In the current study, such chimerism is proposed to explain the lack of predisposing mutations by blood tests, whereas the mutations might be present in tumor tissue.

## Methods

### Patients and clinical assessment

Patients for this study were selected based on the absence of a known familial BRCA mutation found in a first-degree relative. Approval for this study was obtained by the Rush University Medical Center Institutional Review Board. After subjects signed an informed consent form, tumor specimens were obtained from the Department of Pathology, Rush University Medical Center (Chicago, IL) and other respective Pathology Departments of institutions where participants had their cancer surgery. Diagnosis of breast, ovarian or endometrial cancer was obtained from pathology reports and histologic evaluation. Clinical data were established from chart review. Patients were eligible if they were affected by HBOC-associated cancer and had previously tested negative for a known familial mutation. Breast cancer patients under 45 or women with ovarian, fallopian tube, primary peritoneal cancer at any age, any male with breast cancer, and any patient with pancreatic cancer were all considered eligible for this study.

### Isolation of DNA

Hematoxylin and eosin-stained tissue sections cut adjacent to unstained 4 μm were examined by a pathologist. Using the stained slide as a guide, approximately 2mm^2^ of tumor tissue was manually scraped from the slides. The tissue was digested in a solution of 1.0 mg/mL proteinase K (Sigma) in10mM Tris, pH 8.3, 50 mM KCl. Digestions proceeded overnight at 56 ^o^C. The lysate was used directly for analysis.

### Tumor mutation detection

Familial mutations included *BRCA1* 187delAG (a founder mutation in Ashkenazi Jewish population; 2 patients), 1793delA, IVS17+3A>G, 2841G>T, 3109insAA, 5215G>A, 8107G>A, and *BRCA2* 6794 insA, 5645C>A and 6174delT (another founder mutation in Ashkenazi Jewish population). Tumor tissues were tested using sensitive PCR methods to optimize detection. Tissue from families with the *BRCA1* 187delAG mutations were analyzed by amplicon size [47] and pyrosequencing [48]. Amplicon size was also applied to detection of the *BRCA1* 3109 insAA, and *BRCA2* 6794 insA mutations using PCR with fluorescently labeled primers and capillary electrophoresis (Table 1). Fluorescent PCR products were resolved by capillary electrophoresis. Fragment size analysis was performed using GeneMapper Software. Synthetic oligomers (Integrated DNA Technologies, Des Moines, IA) were used as controls for all mutations.

**Table 1:** Mutation primer sequences. Primers used to detect point mutations by sequence specific PCR, Inner primers end on the indicated variant bases. The size of the resulting product of the extended inner primer and its opposite outer primer will indicate the mutation status.

Mutation	Primer Sequences (5’- 3’)	Product Size (bp)
*BRCA1* IVS17+3A>G	• ACTACTCATGTTGTTATGAAAACAGTTG (Forward inner G allele) • GCAAGGTATTCTGTAAAGGTTCTTGGGAT (Reverse inner A allele)\ • TATTTGATTTAATTTCAGATGCTCGTGT (Forward outer primer) • GTCTCGATCTCCTAATCTCGTGATCT (Reverse outer primer)	163, 137
*BRCA2* 8107 A>T	• GAATTTGGGTTTATAATCACTATAGATCGA (Forward inner A allele) • TCCATAGCTGCCAGTTTCCATATCAA (Reverse inner T allele)\ • GGTGTGGATCCAAAGCTTATTTCTAGA (Forward outer primer) • AGGCATCTATTAGCAAATTCCTTAGGAA (Reverse outer primer)	92, 80
*BRCA1* 5215G>A	• AAACAGATGCTGAGTTTGTGTGTGACCA (Forward inner A allele) • CGCAATTCCTAGAAAATATTTCAGTGGCC (Reverse inner G allele)\ • TGACCCCAGAAGAATTTATGCTCGTGTA (Forward outer primer) • TCTAGCCCCCTGAAGATCTTTCTGTCCT (Reverse outer primer)	227, 155

*BRCA1* 187delAG, *BRCA1* 3109 insAA and *BRCA2*, 6794 insT and 6174delT were tested by PCR with fluorescently labeled primers followed by capillary electrophoresis. The 5’ end of one primer for amplification of each gene mutation in the reaction mix described above was covalently bound to fluorescein. Following PCR, amplicons were diluted 1/400 in formamide and separated by capillary electrophoresis. Peak migration compared to controls was used to determine the presence of deletion or insertion mutations.

Detection of the *BRCA1* 187delAG mutation was also performed by pyrosequencing. A 110 bp region covering the *BRCA1* 2 bp deletion was amplified on an ABI9700 Thermal Cycler (ThermoFisher, Waltham, MA). Single strands from the PCR products were isolated and subjected to pyrosequening on a Q24 Pyrosequencer (Qiagen, Inc, Valencia, CA) using primers and sequence to analyze as previously reported [49].

*BRCA1* IVS17+3A>G, and 5215 G>A and *BRCA2* 8107A>T were detected using sequence-specific PCR. Four primers were designed by public access software (http://cedar.genetics.soton.ac.uk/public_html/primer1.html). The four primers (Table 1) were used to amplify the isolated DNA in the following amplification reaction mix: 400 nM inner and outer forward and reverse primers, 1X Taq Gold Buffer (Applied Biosystems), 2.5 mM MgCl_2_, 320 μM dNTP's, 0.25 unit AmpliTaq Gold polymerase (Applied Biosystems) and 5μl template DNA in a total volume of 25 μL. For some assays, primer pairs recognizing the mutant and normal sequences were used in separate reactions. The two inner primers from each group of four are designed to end on either the normal or mutated base. If the normal base is present, the inner primer ending with the complement to the normal base will be extended with one of the outer primers to yield a product of the indicated size. For example detection of the *BRCA1* IVS17+3A>G, the normal sequence will yield a product of 137 bp. The other inner primer will yield a product of 163 bp if the mutant base is present in the template (Table 1).

*BRCA1* 2841G>T and *BRCA2* 5645C>A were analyzed by dye terminator sequencing (Illumina). 50 ng of tumor DNA was subjected to a first round of target selection by PCR, followed by a second round of indexing. Pooled samples were cleaned with magnetic beads, and 12 pmol was loaded into the Illumina MiSeq. Sequence results were screened for coverage (500X) and variant frequency.

#### Results

The eleven patients studied carried diagnoses of infiltrating ductal carcinoma, ductal carcinoma in situ, invasive lobular carcinoma, ovarian adenocarcinoma as well as endometrial and primary peritoneal carcinoma. Patient ages at diagnosis ranged from 26 to 66 years. None of the eleven patient tumors tested displayed the familial *BRCA* mutation alleles (Table 2). In addition to testing for g*BRCA* mutations, four patients underwent gene panel testing and were negative for mutations in other cancer-associated genes. Patients 2 and 9 underwent gene panel testing that included 23 genes: *ATM*, *BARD1*, *BRCA1*, *BRCA2*, *BRIP1*, *CDH*, *CHEK2*, *EPCAM*, *MLH1*, *MRE11A*, *MSH2*, *MSH6*, *MUTYH*, *NFN*, *NF1*, *PALB2*, *PMS2*, *PTEN*, *RAD50*, *RAD51C*, *RAD51D*, *STK11*, and *TP53*. Patient 3 underwent a more comprehensive gene panel test due to her significant personal and family history of cancer that included 49 genes: *APC*, *ATM*, *BAP1*, *BARD1*, *BRCA1*, *BRCA2*, *BRIP1*, *BMPR1A*, *CDH1*, *CDK4*, *CDKN2A*, *CHEK2*, *EPCAM FH*, *FLCN*, *GREM1*, *MAX*, *MEN1*, *MET*, *MITF*, *MLH1*, *MRE11A*, *MSH2*, *MSH6*, *MUTYH*, *NBN*, *NF1*, *PALB2*, *PMS2*, *POLD1*, *POLE*, *PTEN*, *RAD50*, *RAD51C*, *RAD51D*, *RET*, *SDHA*, *SDHAF2*, *SDHB*, *SDHC*, *SDHD*, *SMAD4*, *SMARCA4*, *STK11*, *TMEM127*, *TP53*, *TSC1*, *TSC2*, and *VHL*. Patient 7 underwent a gene panel specific to breast cancer risk that included 14 genes: *ATM*, *BARD1*, *BRIP1*, *CDH1*, *CHEK2*, *MRE11A*, *MUTYH*, *NBN*, *PALB2*, *PTEN*, *RAD50*, *RAD51C*, *STK11*, and *TP53*. Patient 10 underwent Lynch syndrome testing (*MLH1*, *MSH2*, and *MSH6*) in addition to *BRCA1/2* due to a personal history of early onset endometrial cancer. None of the patients had a pathogenic mutation in the other genes tested.

**Table 2:** Patient clinical characteristics and family history.

Patient	Age	Cancer	Stage	Ethnicity	Family History	Family Mutation	Patient Mutation identified on additional blood testing	Panel Testing
1	47	Infiltrating ductal carcinoma, ER/PR-, HER2+	2	Ashkenazi	• Mother, breast (40s), ovarian (?): BRCA+ • Sister, breast (47): BRCA+ • MGF, stomach • 2 first cousins: BRCA+	BRCA1 187delAG		No
2	58	Ovarian cancer	1	Mexican	• Sister, breast (55), ovarian (56): BRCA+ • Sister, breast (40) • Sister, leiomyosarcoma (67) Brother, lymphoma (72) • Brother, melanoma (48) • M aunt, breast (68) Niece (sister), breast (22)	BRCA1 1793delA		No
3	57	Primary peritoneal cancer	4	German, Irish	• Mother, breast (42) • Sister, breast (36), ovarian (47): BRCA+ • Sister, breast (39), ovarian (56) • Brother, LGL leukemia (48) • MGF, breast • Niece (brother), breast (25)	BRCA1 2841G>T	VUS (MSH2- p.S87C)	Yes
4	57	Ductal carcinoma in situ	0	Ashkenazi	• Mother, colon (61) Father, colon (73) • Sister, breast (61): BRCA+ M uncle, colon • P cousin, breast, ovarian (60)	BRCA2 6174delT		No
5	66	Ductal carcinoma in situ	0	Ashkenazi	• Mother, breast (70) • Sister, ovarian (67), breast (69), lung (71): BRCA+ • Niece (sister), ovarian (47): BRCA+ • Unaffected niece (sister): BRCA+	BRCA1 187delAG		No
6	61	ILC, ER/PR+, HER2-	1	Danish, Polish, English, Welsh, German	• Sister, ovarian (62): BRCA+ • Father, unknown primary (70s) • PGF, colon • Aunt, breast (80s)	BRCA1 5215G>A		No
7	51	Infiltrating ductal carcinoma, ER/PR+, HER2-	2	Unknown	• Father, prostate: BRCA+ • Sister, breast, BRCA+ • M cousin, colon (48) • M cousin, breast (50) • PGM, breast • P great aunt, breast • P 2^nd^ cousin, ovarian • P greatGM, GI cancer	BRCA1 8107G>A	VUS (ATM- c.496+4T>C)	Yes
8	51	Infiltrating ductal carcinoma	U	German, Polish	• Mother, ovarian (64) Unaffected sister: BRCA+ • MGM, ovarian/stomach • M aunt, ovarian (60s) P uncle, kidney	BRCA1 IVS17+3A>G		No
9	61, 73	Infiltrating ductal carcinoma and primary peritoneal	U	Unknown	• Mother, lung (89) • Father, breast, larynx • Sister, breast (42), breast (62): BRCA+ • Brother, pancreatic (62) • PGM, ovarian (51) • P cousin, pancreatic (64)	BRCA2 5645C>A		Yes
10	43	Endometrial cancer	3	Filipino	• Father, colon (60s) • Unaffected sister: BRCA+ • Sister, ovarian (40) • M aunt, breast (60s) • M cousin, breast (29): BRCA- • M cousin, breast (50) • P aunt, sarcoma (40s) • P uncle, lung (60s)	BRCA2 6794insA		No, but had additional testing for Lynch syndrome
11	26	Ovarian cancer	3	Unknown	• Mother, breast (38) • Unaffected brother: BRCA+ • MGM, ovarian	BRCA1 3109insAA		No

U—unknown stage

**Fig 2:**
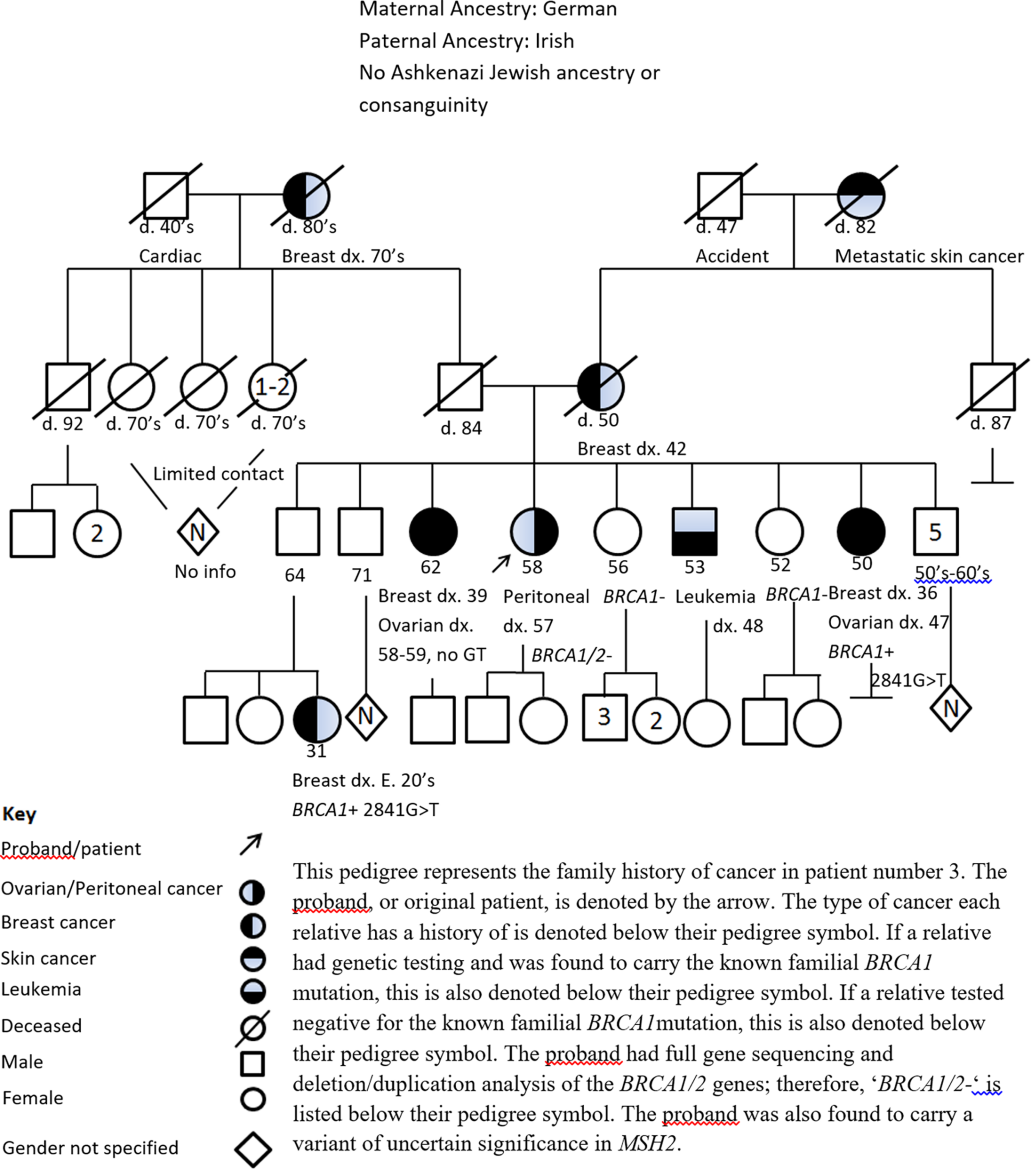
Patient 3 pedigree. This pedigree represents the family history of cancer in patient number 3. The proband, or original patient, is denoted by the arrow. The type of cancer each relative has a history of is denoted below their pedigree symbol. If a relative had genetic testing and was found to carry the known familial *BRCA1* mutation, this is also denoted below their pedigree symbol. If a relative tested negative for the known familial *BRCA1*mutation, this is also denoted below their pedigree symbol. The proband had full gene sequencing and deletion/duplication analysis of the *BRCA1/2* genes; therefore, ‘*BRCA1/2-*‘ is listed below their pedigree symbol. The proband was also found to carry a variant of uncertain significance in *MSH2*.

**Fig 3:**
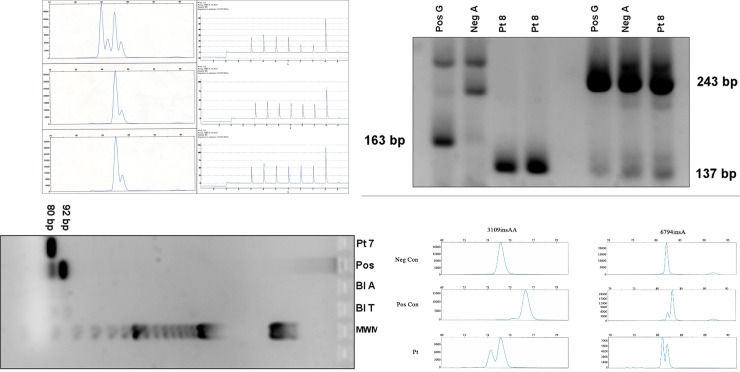
Molecular testing in tumor tissue. **A.** The *BRCA1* 187del AG deletion heterozygote (families 1 and 5) is detected as an n-2 product by capillary electrophoresis (left) and by an indicative peak pattern by pyrosequencing (top right). Neither the deletion product nor the mutant peak pattern was detected in the patient tumors (bottom panels). **B.** The heterozygote detected as a 163 bp product by gel electrophoresis. The synthetic oligomer carrying the mutation confirmed the detection of the mutation by mutation sequence specific primers. This band is not present in the negative control nor family 8 DNA (left four lanes). The 137 bp band specific to the normal A allele was detected by primers specific to that allele in the patient sample. Tumor DNA tested for the familial mutation 5215G>A gave similar results (not shown). **C.** The *BRCA2* 8107 A→T mutation was tested by sequence-specific PCR. The 92 bp product (T allele, positive) is not present in the patient’s tissue where only the A allele (80bp) is observed. Reagent blanks for the A and T allele primer sets (Bl A, Bl T) are shown. **D.** *BRCA1* 3109 insAA (left), and *BRCA2* 6794 insA (patients 10 and 11, respectively; right) mutation analysis by PCR-capillary electrophoresis. Amplified products from DNA without (negative control, top) and with (positive control, middle panels) demonstrate the expected right shift in migration for the *BRCA1* 3109 insAA n+2 product (76 bp) and the *BRCA2* 6794 insA n+1 product (82 bp)(bottom panels). Patient samples show an unexpected left shift (n-1) product.

Patient 3 underwent genetic counseling following her sister was found to have a deleterious *BRCA*1 mutation and was found to be a non-carrier. Despite these findings, she underwent prophylactic bilateral salpingo-oophorectomy with hysterectomy and pathology was negative for occult malignancy. She was diagnosed with primary peritoneal carcinoma 12 months after her prophylactic surgery. See Fig 2 for this patient’s pedigree. Families of patients 1 and 5 carried the *BRCA1* 187delAG mutation. Assessment of the tumor tissue from the two phenocopy patients by PCR/capillary electrophoresis and by pyrosequencing revealed no evidence of the mutation (Fig 3A).

Testing for the *BRCA1* IVS17+3A>G, 5215 G>A and 8107A>T mutations (tumors from patients 6, 7, 8) was performed by sequence-specific PCR. Peak height patterns of the resulting pyrograms were analyzed to detect the 187 delAG mutations (Fig 3B). In all three cases, the mutation would be detected as the intermediate sized band of three bands on the gel. The *BRCA2* 8107 A>T mutation (tumor from patient 7) was tested using sequence-specific PCR (Fig 3C). This method was originally designed as a multiplex PCR, however, due to primer competition, individual primer sets for mutant and wild type alleles were used in separate reaction mixes. The amplicons of the two separate reactions for each sample were then mixed and loaded into a single well for electrophoresis. While the product of the A allele was present in the patient sample, the 92 bp T allele seen in the positive control was not. The results in Fig 3B and 3C show no detection of mutations in the phenocopy tumor tissue.

*BRCA1* 3109 insAA and *BRCA2*, 6794 insA (tumors from patients 10, 11) were assayed using PCR with fluorescently labeled primers and capillary electrophoresis (Fig 3D). These analyses yielded some equivocal results. As shown in Fig 3D, PCR products from the patient DNA migrated in a pattern different from the positive controls (shifted 1–2 bp to the right from the negative control from 74 to 75 or 82 to 84 bp). Migration was partially consistent with the negative control, however, an additional (n-1) product was apparent in both specimens. Subsequent reversible dye terminator sequencing (Illumina) did not detect the respective mutations in either sample.

## Discussion

The current study addresses the presence of the disease phenocopy in the absence of a familial gene mutation. Genetic testing is most often performed on blood which is an easily accessible, abundant source of high quality DNA. Blood is thought to provide a genotype representative to all other tissues. Rare genetic events early in embryonic development could theoretically result in an individual with different genotypes in different tissues (chimera). In this case, mutations in tissue may not be present in blood. If these mutations are cancer-associated, risk of malignancies in the tissue would be increased, without the presence of the genetic mutation in the blood.

Our study included patients with ovarian, primary peritoneal and endometrial cancers which are uncommon in the general population and less likely to develop sporadically, especially, in families with known HBOC syndrome suggesting an underlying cause for these phenocopies. We tested DNA from eleven tumors from women who come from families carrying *BRCA1* and *BRCA2* mutations, but who do not carry the familial mutation themselves (phenocopies) as defined by blood testing. Phenocopies are rare but their exact incidence is unknown. Their personal and family history of cancer may suggest a genetic cause for the phenotype and we postulated that they may represent a genetically chimeric state. To investigate this, we looked for the familial mutation for each patient in the tumor tissue. Tumor tissue is not commonly tested for familial mutations. We used highly sensitive PCR methods to determine if the familial mutations might be present in the affected tissues. None of the eleven samples displayed these mutations. This observation does not support that chimerism is responsible for the phenocopies.

This result is concordant with the result of a recent study by Azzollini *et al*. However, there are significant differences between the studies. Specifically, in their study [49], the authors test the hypothesis of spontaneous reversal of germline *BRCA* mutations to the wild-type in blood cells which may account for the negative result of genetic testing performed on blood. To our knowledge, this phenomenon was observed in malignant tissue from germline *BRCA* carriers not randomly, but rather due to their exposure to cytotoxic chemotherapy (most commonly, platinum agents) [50–52] This reversal is believed to account for developing platinum resistance in some *gBRCA* carriers with cancer treated with Carboplatin. In addition, spontaneous reversal of the germline mutations is unlikely to lead to the wild-type gene sequence in the majority of white blood cells. Since genetic testing has a sensitivity of 99% [53] for detection of a mutation, the germline mutation present even in 1% of blood cells should have been still detected. In contrast to the study Azzollini *et al*, our study analyzed only tumor samples from *BRCA* phenocopies but not other tissues. Also, for the purpose of our study, *BRCA* phenocopies were defined as the first-degree relatives of the known *BRCA* carriers as opposed to less closely related family members.

Finally, our hypothesis is in fact quite different from the hypothesis of Azzollini *et al* as it is based on the suggestion that an individual can develop a tumor originating from the cells of another individual. Cancers, allogeneic in origin, have been observed in other species such as an aggressive devil facial tumor disease in Tasmanian devils and an indolent transmissible venereal tumor in dogs [54]. In humans, rare cases of allogeneic tumors were observed in infants born to mothers with a metastatic malignancy (maternal tumors) [55] and in transplant recipients with suppressed immunity (tumors of donor origin) [56–62]. Even more intriguing is the recent research on the role of fetal-maternal and maternal-fetal microchimerism in human cancers [63–66]. According to these studies, fetal cells are found in maternal tumors many years after pregnancy as low-abundancy cells. Their role in carcinogenesis remains unknown. Some studies suggest their protective role in breast [64, 67] and other maternal cancers, while others point towards their unfavorable role in promoting tumorigenesis [66]. However, so far no studies showed that fetal cells can directly give rise to cancer by forming a malignant clone in a mother, although some research suggests that it is possible [68]. Given these considerations, we were planning to test our study participants for chimerism using STR-based (Short tandem repeat genotyping) methods had we demonstrated the presence of the mutation from the family member in their tumor.

Potentially, germline mutations in other cancer-associated genes may account for cancers in *BRCA* non-carriers. However, in our study, some of the participants underwent multi-gene panel testing looking for mutations in other genes commonly associated with predisposition to breast and ovarian cancers. We did not find any pathogenic germline mutations to explain the HBOC phenotype in *BRCA* phenocopies. Even though we found two variants of unknown significance (VUS) in the *ATM* and *MSH2* genes, they are likely to be eventually re-classified into benign polymorphisms like most of other VUSes. Pathogenic mutations in the *MSH2* gene are associated with Lynch syndrome. Lynch syndrome is known to cause colorectal, endometrial, gastric, ovarian, and urinary tract cancers. Lynch syndrome does not currently have a definitive link to increased risk for breast cancer [69]. Case 3 who had a *MSH2* VUS does not have a family history consistent with Lynch syndrome which makes the VUS finding less concerning. Mutations in the *ATM* gene are associated with an increased risk for many of the same cancers associated with *BRCA* mutations, such as breast, pancreatic, and prostate. While case 7 has a personal and family history of cancers that could be associated with a germline *ATM* mutation, most affected relatives tested positive for the familial *BRCA* mutation, explaining the cancer history in these individuals.

It has been observed that individuals who test negative for a known familial mutation in other cancer-associated genes may still be at an increased risk to develop certain types of cancer. One example is the *CHEK2* gene. It has been well-established in multiple studies that germline *CHEK2* mutations are associated with an increased risk for breast, colon and other cancers. Current research has shown discrepancies in the level of breast cancer risk in families with *CHEK2* mutations suggesting that there are additional factors that influence the risk of breast cancer in these families in addition to the *CHEK2* mutation. Therefore, even when a person tests negative for a known familial *CHEK2* mutation previously identified in a blood relative, they are still considered to be at an increased risk to develop breast cancer. On the other hand, in Lynch syndrome, the study of non-carrier relatives did not demonstrate an increased cancer risk [70].

Aside from other pathogenic gene mutations in other high or moderate penetrance genes, single nucleotide polymorphisms (SNPs) could be an additional explanation for *BRCA* phenocopies. A SNP occurs when a single nucleotide differs from the nucleotide seen at this locus in the genome of the general population [71]. SNPs are normal and occur approximately once in every 300 nucleotides. They are population-specific. Typically they are thought to have no effect on a person’s health. However, more recent studies have shown a potential link between certain SNPs and an increased risk of breast cancer. Combinations of SNPs have been proposed to assess a woman's risk of breast cancer if she has a family history of breast cancer but no identifiable pathogenic mutation in a cancer-associated gene [72–75]. Penetrance of g*BRCA* mutations vary in families and populations. Some SNPs have been reported to modify cancer risk in g*BRCA* mutation carriers [76–77]. It is possible that the same SNPs that increase cancer risk in g*BRCA* carriers can increase the cancer risk in their non-carrier relatives and thus, account for the phenomenon of *BRCA* phenocopies.

The *BRCA* phenocopy phenomenon undermines the value of genetic testing for HBOC for some health care providers and patients alike. Accordingly, some women from the *BRCA* positive families affected by HBOC make the decision for prophylactic surgery even in the absence of the known familial mutation. Our case 3 is one of the most striking examples. This patient decided to undergo risk-reducing bilateral-oophorectomy and hysterectomy despite of the negative blood test for the familial mutation. Nonetheless, she was diagnosed with primary peritoneal carcinoma within a year after the prophylactic surgery.

## Conclusion

Hereditary breast and ovarian cancer syndrome most frequently occur through inheritance of mutations in the *BRCA1* and *BRCA2* genes. Non-carriers in a family with a known mutation in either gene could be at higher risk for cancer but the current recommendations are for that of the general population. Our hypothesis and the hypothesis of Azzollini *et al*, presumed a link between a familial *BRCA* mutation and HBOC in *BRCA* phenocopies, albeit through different mechanisms (chimerism and mosaicism, respectively). Although both studies were negative, which strengthens the conclusion that there is no association between the familial mutation and cancer development in *BRCA* phenocopies, full tumor sequencing is now possible on tumor cells which may provide further insight into the pathogenesis of the tumors. Currently, the ultimate cause of this phenomenon of phenocopies remains unknown. Further investigation on these tumor specimens with comprehensive sequence analysis of additional five hundred cancer-associated genes by next-generation sequencing is underway. We believe that different approaches to study phenocopies are warranted including investigation of genome-wide associations (GWAS) and SNPs.
